# Non-random Escape Pathways from a Broadly Neutralizing Human Monoclonal Antibody Map to a Highly Conserved Region on the Hepatitis C Virus E2 Glycoprotein Encompassing Amino Acids 412–423

**DOI:** 10.1371/journal.ppat.1004297

**Published:** 2014-08-14

**Authors:** Zhen-yong Keck, Allan G. N. Angus, Wenyan Wang, Patrick Lau, Yong Wang, Derek Gatherer, Arvind H. Patel, Steven K. H. Foung

**Affiliations:** 1 Department of Pathology, Stanford University School of Medicine, Stanford, California, United States of America; 2 MRC – University of Glasgow Centre for Virus Research, Glasgow, United Kingdom; 3 Division of Biomedical and Life Sciences, Lancaster University, Lancaster, United Kingdom; Nationwide Children's Hospital, United States of America

## Abstract

A challenge for hepatitis C virus (HCV) vaccine development is to define epitopes that are able to elicit protective antibodies against this highly diverse virus. The E2 glycoprotein region located at residues 412–423 is conserved and antibodies to 412–423 have broadly neutralizing activities. However, an adaptive mutation, N417S, is associated with a glycan shift in a variant that cannot be neutralized by a murine but by human monoclonal antibodies (HMAbs) against 412–423. To determine whether HCV escapes from these antibodies, we analyzed variants that emerged when cell culture infectious HCV virions (HCVcc) were passaged under increasing concentrations of a specific HMAb, HC33.1. Multiple nonrandom escape pathways were identified. Two pathways occurred in the context of an N-glycan shift mutation at N417T. At low antibody concentrations, substitutions of two residues outside of the epitope, N434D and K610R, led to variants having improved in vitro viral fitness and reduced sensitivity to HC33.1 binding and neutralization. At moderate concentrations, a S419N mutation occurred within 412–423 in escape variants that have greatly reduced sensitivity to HC33.1 but compromised viral fitness. Importantly, the variants generated from these pathways differed in their stability. N434D and K610R-associated variants were stable and became dominant as the virions were passaged. The S419N mutation reverted back to N419S when immune pressure was reduced by removing HC33.1. At high antibody concentrations, a mutation at L413I was observed in variants that were resistant to HC33.1 neutralization. Collectively, the combination of multiple escape pathways enabled the virus to persist under a wide range of antibody concentrations. Moreover, these findings pose a different challenge to vaccine development beyond the identification of highly conserved epitopes. It will be necessary for a vaccine to induce high potency antibodies that prevent the formation of escape variants, which can co-exist with lower potency or levels of neutralizing activities.

## Author Summary

An effective hepatitis C virus (HCV) vaccine will require information on epitopes that are responsible for protective antibodies against this highly diverse virus. A region known to be highly conserved and responsible for broadly neutralizing antibodies is located on the E2 glycoprotein at 412–423. To test whether HCV can escape from human antibodies against this region, infectious virus was passaged in culture in increasing concentrations of a human monoclonal antibody to 412–423. Multiple pathways of viral escape were identified at different levels of antibody concentrations. Some of the escape virions were stable and were more robust than wild-type virus. Other escape virions were unstable and had compromised in vitro viral fitness. Collectively, these findings underscore the difficulties in HCV vaccine development and the need to induce high potency antibodies not associated with viral escape.

## Introduction

Infection with hepatitis C virus (HCV) is a leading cause of chronic hepatitis, cirrhosis and hepatocellular carcinoma. The World Health Organization estimates an annual increase in the global burden by 3–4 million new infections [1]. Encouragingly for patients, advances in *in vitro* and *in vivo* HCV infection systems and increased understanding of HCV virology have led to the development of many promising HCV-specific direct acting antivirals (DAA) [2]–[6]. However, the high costs of DAA will limit their access to the large majority of HCV infected patients living in countries with limited resources. There is clearly a need for a preventive HCV vaccine. Humoral immunity is the primary correlate of protection for most preventive vaccines, as shown for smallpox and other DNA viruses. For HCV, cumulative evidence supports the importance of virus neutralizing antibodies to facilitate clearance. Chimpanzee studies showed that protection from an infectious HCV inoculum is correlated with HCV-specific antibody titers blocking infection of target cells with pseudotyped retroviral particles expressing HCV E1E2 glycoproteins (HCVpp) [7]. Neutralizing antibody response measured via HCVpp has been associated with control of infection in single source outbreaks of acute HCV infections [8], [9], and in a study of active injection drug users (IDUs) [10]. While only 25% of IDUs in this study cleared primary HCV infection, 83% cleared subsequent re-infection episodes, and clearance was associated with cross-reactive neutralizing antibodies. In addition, antibodies to HCV E2 prevent infection in a human liver-mouse chimeric model [11], [12]. Finally, an immunocompetent humanized mouse model for HCV exhibited a robust antibody response to a recombinant vaccinia virus expressing HCV proteins that protected against an infectious HCV challenge in some animals that correlated with the serum level of E2 antibodies [13]. A key challenge for vaccine design is to overcome the genetic diversity of the virus. This will require information on conserved epitopes mediating virus neutralization and on the mechanisms of HCV escape from the humoral immune response.

HCV is a positive-strand RNA virus encoding a polyprotein that undergoes proteolytic cleavage to 10 polypeptides, each with distinct functions. The two envelope glycoproteins, E1 and E2, form a heterodimer that mediates viral entry [14]–[16] through interactions with cellular receptors (reviewed in [17]), and are the natural targets for neutralizing antibodies. Both proteins are highly glycosylated that partly shields the virus from neutralizing antibodies [18]–[21]. The genes encoding E1 and E2 are the most variable in the HCV genome. The hypervariable region one (HVR1) in E2 is immunodominant and infected individuals develop isolate-specific neutralizing antibodies against this region throughout the course of their infections [22], [23]. These antibodies provide little protection since the HVR1 sequence continuously evolves in response to pressure exerted by HVR1-specific neutralizing antibodies leading to viral escape [23], [24]. An effective HCV vaccine will need to include conserved epitopes that are able to elicit broadly neutralizing antibodies. Much effort has been devoted to the identification of conserved regions mediating virus neutralization through the isolation and characterization of human monoclonal antibodies (HMAbs) from the B cells of HCV-infected individuals and of murine monoclonal antibodies from recombinant E2 glycoprotein immunized mice. The focus has been primarily on E2 since this viral structure interacts with HCV co-receptors and is more immunogenic than E1. Studies with HMAbs to E2 have led to the delineation of at least six distinct clusters of overlapping linear and nonlinear epitopes, designated as antigenic domains A-E [25]–[28]. Many of these HMAbs from different laboratories are to overlapping epitopes, which can be grouped in one cluster, antigenic domain B. Of concern, some domain B antibodies do not neutralize all HCV genotypes, which is indicative of escape [29]. Single amino acid substitutions also can lead to viral escape with other domain B antibodies [30], [31], similar to escape from antibodies against the HVR1 [24]. There are three patterns of viral escape that are observed when infectious cell culture virions (HCVcc) are grown in the presence of neutralizing domain B antibodies [32]. Of the three tested domain B HMAbs, one led to escape mutant viruses without affecting in vitro viral fitness; a second led to escape but with compromised viral fitness; and a third led to complete virus elimination at a critical antibody concentration without escape mutants. Sequence analysis of escape mutants revealed a conserved region, amino acid (aa) 529–535, and a region, aa 425–443, on E2 that appears to be associated with escape mutations [32].

Immediately downstream of HVR1 is a cluster of overlapping linear epitopes that are highly conserved across all HCV genotypes and subtypes, encompassing aa 412–423, but are of low immunogenicity in population studies [33], [34]. A number of broadly neutralizing monoclonal antibodies targeting this region have been isolated from experimentally immunized mice [16], [35]–[37] and a human monoclonal antibody, designated as HCV1, in a transgenic mouse [38]. Their precise contact residues have been resolved by direct crystal structure of E2 peptides in complex with two of these antibodies, AP33 and HCV1 [39]–[41]. Other studies have also established that this region is involved in virus binding to the HCV co-receptor, CD81 [37], [42], which explains why this region is highly conserved in order to preserve essential viral functions. Thus, antibodies to this region have held great promise for immunotherapy and vaccine development. However, the Asn at 417 is an N-linked glycosylation site that shields this conserved region from being fully exposed to neutralizing antibodies by reducing epitope access [18]–[21]. An adaptive mutation N417S that leads to a glycan shift upstream to N415 blocks virus neutralization by AP33 and HCV1 [43]–[45]. The N-glycan shift at N417 occurs frequently in passaged HCVcc. The shift occurs in the absence of selection by neutralizing antibodies targeting this region, in the presence of neutralizing antibodies targeting different regions or in the presence of a non-HCV HMAb [28], [32].

We recently isolated a panel of HMAbs to aa 412–423 [25]. Surprisingly, cell culture adapted 2a JFH1 HCVcc, containing mostly glycan shifted HCVcc at N417S and a minor population of wild-type (wt) HCVcc, displayed an increased sensitivity to neutralization by these HMAbs, in contrast to the lack of neutralization by a murine monoclonal antibody. This raised questions whether and how HCV can escape from human antibodies directed against aa 412–423, particularly because the mutation leading to an N-glycan shift from 417 to 415 does not lead to viral escape, but to an increase in sensitivity to these antibodies. This report addresses these questions by assessing viral evolution in the presence of a HMAb against aa 412–423, designated as HC33.1. Sequence analyses of variants obtained at different time points when 2a HCVcc was co-cultured with HC33.1, from low to high antibody concentrations, revealed multiple patterns of mutations. At low antibody concentrations, mutations occurred outside of aa 412–423 in combination with an N-glycan shift mutation at N417T. These variants exhibited improved viral fitness and reduced sensitivity to HC33.1 binding and neutralization. At moderate antibody concentrations, a mutation was observed within the conserved aa 412–423 region at residue 419 in escape variants having compromised fitness and greater reduction in sensitivity to HC33.1. Interestingly, when HC33.1 was removed, the 419 mutation-associated variants rapidly disappeared and the variants that emerged contained the wild-type residue at this position. At high antibody concentrations, a mutation at 413 was observed in variants that were completely resistant to HC33.1 neutralization. Taken together, multiple pathways are involved in viral escape from a single antibody that appear to be concentration dependent, and associated with and without compromised in vitro viral fitness in escape variants.

## Results

### HMAbs and a mouse monoclonal antibody to aa 412–423 have different neutralization profiles

**Figure 1:**
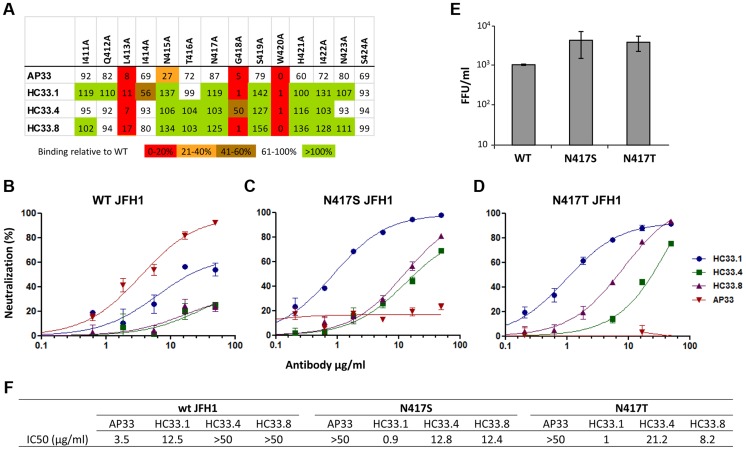
Human and mouse antibodies against amino acid 412–423 have different neutralization profiles. (**A**) Epitope alignment. Epitopes of three HMAbs: HC33.1; HC33.4 and HC33.8 are compared with murine MAb AP33. Recombinant E1E2 mutant proteins were expressed in 293T cells and cell lysates were analyzed by ELISA. Individual protein expression was normalized by binding of CBH-17, an anti-HCV E2 HMAb to a linear epitope [25], [47], [51]. Red indicates 0–20%, orange 21–40%, brown 41–60%, white 61–100% and green >100% binding, when the residue was replaced by alanine, relative to binding to wt. Dose-dependent neutralization of (**B**) wt JFH-1 HCVcc, (**C**) HCVcc variant bearing N417S mutation and (**D**) bearing N417T mutation were performed by SEAP reporter assay. (**B, C, D**) Either wt HCVcc or variant HCVcc was incubated with HC33.1 or AP33, at concentrations ranging from 0.1 to 50 µg/ml, prior to infecting the Huh7J-20 cells. Virus infectivity levels were determined by measurement of the SEAP activity released into the medium. (**F**) Antibody concentration (µg/ml) required to reach 50% neutralization (IC_50_) for each antibody is summarized. (**E**) The effect of N417S or N417T mutation on in vitro viral fitness as measured by focus forming assay at an MOI of 0.1. (**B–E**) Each assay was performed in triplicates and data are shown as percent neutralization, the mean of two experiments ±SD.

Employing AP33 as the prototype antibody to this region [16], [35]–[37], [43], epitope mapping of this and three HMAbs to aa 412–423, HC33.1, HC33.4 and HC33.8 (designated as antigenic domain E), revealed shared contact residues at L413, G418 and W420, as determined by <20% binding to a panel of alanine substitution H77C E1E2 mutants (Fig. 1A) [25]. No contact residues were identified between aa 425–443 with the HC33 HMAbs (data not shown). They differed at residue 415, in which a N415A mutation led to AP33 binding reduction of 73% and the HC33 HMAbs having no binding reduction. To define the different neutralization profiles of AP33 and HC33 HMAbs against glycan shifted HCVcc variants, JFH1 E1E2 plasmids were constructed to contain N417S or N417T. The N417S mutation has been shown to be an adaptive mutation [45] and the N417T change was observed in the HC33.1 selection studies (see below). Both substitutions resulted in an N-glycan shift from residue 417 to N415. Wild type and variant HCVcc bearing N417S or N417T were produced, and dose-dependent neutralization was measured with AP33 and the three antigenic domain E HMAbs (Fig. 1B–1D). AP33 and HC33.1 neutralized wt HCVcc, which was poorly neutralized by HC33.4 and HC33.8 (Fig. 1B). AP33 had a higher potency (IC_50_ at 3.5 µg/ml) than HC33.1 (IC_50_ at 12.5 µg/ml) (Fig. 1F). The data is consistent with previous findings of AP33 having high neutralizing potencies against different HCV genotypes [37]. In contrast, AP33 (up to 50 µg/ml) failed to neutralize the N417S and N417T HCVcc variants (Fig. 1C, 1D and 1F); whereas these variants remained sensitive to HC33.1, HC33.4 and HC33.8. The neutralization potencies of the three domain E antibodies significantly improved and their IC_50_ values ranged 0.9 to 21.2 µg/ml. HC33.1 IC_50_ improved by over tenfold against both N417S/T variants compared to wt HCVcc. Of note is that the contact residues for the HCV1 HMAb, isolated from a transgenic mouse that was challenged with recombinant E2 proteins, are similar to the HC33 antibodies, involving 413 and 420 but with a 20 percent reduction at 418 [38]. Yet escape variants for HCV1 have been documented to include N417T/S mutations [44], [46]. These results demonstrate the difference between previously isolated antibodies to aa 412–423 and the HC33 HMAbs that are elicited in response to viral infection undergoing an N-glycan shift from 417 to 415. Moreover, the increased potencies of the antigenic domain E HMAbs to glycan shifted virions confirm the importance of the N-glycan at 417 in shielding aa 412–423 from neutralizing human antibodies [18]–[21].

The relative in vitro viral fitness of N417S and N417T HCVcc variants was compared to wt by determining the virus yield at a low multiplicity of infection (MOI = 0.1) after 96 hours post infection. Both N417S (4.1) and N417T (3.7) had approximately four fold higher viral yields than wt HCVcc (Fig. 1E). Statistical analysis found both N417S (*P* = <0.040) and N417T (*P* = <0.009) to be significantly higher than wt HCVcc. The increased fitness is in agreement with previous findings that the glycan shift associated with N417S is a spontaneous adaptive mutation that confers greater viral fitness [45]. However, the previous study found the increase to be statistically not significant (*P* = <0.063; [45]). The *P* values between the two sets are marginally different and within experimental fluctuations. The findings also show that the residue at 417 is polymorphic, N417, S417 or T417, with the variants having advantages of greater in vitro viral fitness and resistant to neutralization to some antibodies to this region, e.g., AP33 and HCV1 [44]–[46]. But this glycan shift leads to greater susceptibility to other human antibodies to aa 412–423, and to antigenic domain B [45].

### HCV evolution in the presence of increasing concentration of HC33.1

**Figure 2:**
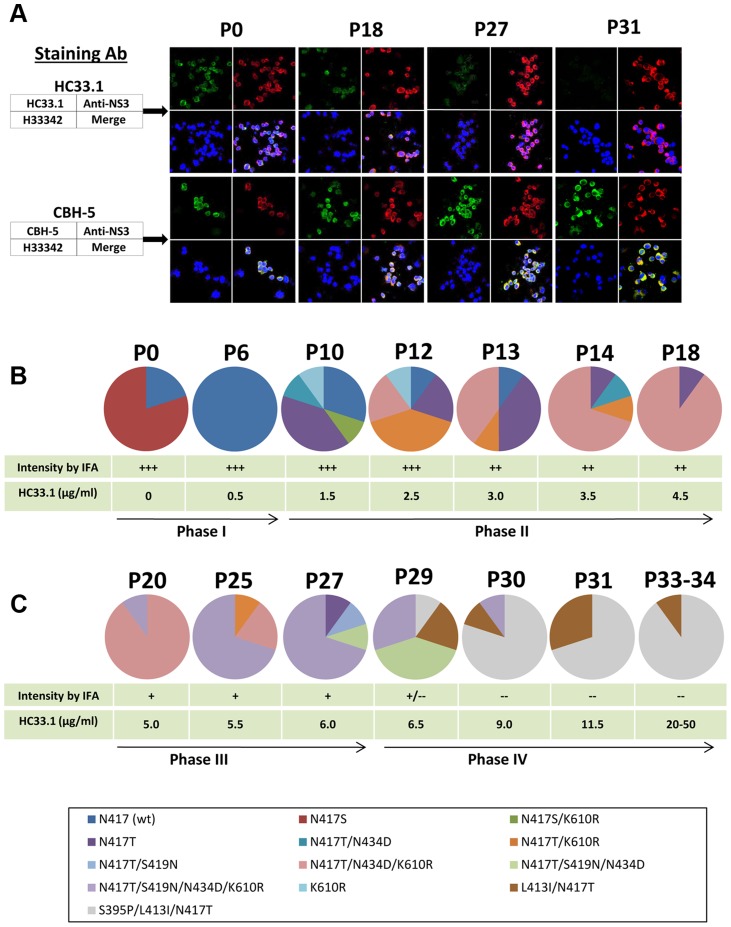
Viral evolution in the presence of increasing HMAb HC33.1 concentrations. (**A**) Dual antibody immunofluorescence staining of Huh7.5 cells infected with JFH1 2a HCVcc during multiple passages in increasing concentration of HC33.1. IFA is shown for P0, P18, P27 and P31. HCV E2 glycoprotein was stained with HC33.1 under which viral escape variants were selected (green, upper set of panels), or with CBH-5, a neutralizing domain B HMAb that does not share the same epitope with HC33.1 on E2 (green, lower set of panels). Total virus-infected cells were stained with anti-NS3 antibody labeled with Alexa-594 (red). The cells were counterstained with Hoechst nuclear stain H33342 (blue). The captured images were superimposed (merge). (**B and C**) Sequence analysis of escape variants in increasing concentrations of HC33.1. Circle graph represents the change in composition of variants from selected passages, as indicated on the top, P0–P34. Specific variants are color coded as indicated in the legend. The table presents the corresponding HC33.1 concentration and the relative intensity in IFA binding by HC33.1. The arrow line divides the viral evolution into four phases (Phase I–IV), based on the appearance in each phase of a variant(s) bearing a specific mutation that became dominant. Viral RNA in the corresponding cell culture supernatants were analyzed by single colony sequencing following RT-PCR amplifications.

Escape from antibody-mediated virus neutralization occurs by mutations at contact residues within the cognate epitope of the virus neutralizing antibody [28], [32]. However, alanine substitution at each of the three contact residues of HC33.1 at L413, G418 or W420 completely abolished HCVpp infection [42], suggesting that mutations at these contact residues probably will not be an escape mechanism from human antibodies to aa 412–423 on E2. Either the virus cannot escape from antigenic domain E antibodies or that escape under immune selection pressure is by a different pathway. This was studied by an *in vitro* antibody-virus co-culture protocol that mimics the evolution of viral antigenic determinants under immune pressure in humans. The antibody-virus co-culture system with a 2a HCVcc isolate identified the same escape mutation for an antigenic domain B antibody as observed in an genotype 1a infected individual [28], [30]–[32]. Similar findings have been reported with other escape mutations that are the same in co-culture studies with a different 2a HCVcc isolate and observed in clinical studies with HCV genotype 1a infected individuals [43], [44]. Thus, we believe that antibody-virus co-culture systems identify escape variants that are broadly applicable to other genotypes/isolates. Extracellular JFH1 HCVcc was passaged in the presence of HC33.1 in increasing antibody concentrations. CBH-2, an antigenic domain B HMAb, was used as a positive (escape) control and R04, an isotype-matched HMAb to CMV was used as negative control. The expectation was that mutations at contact residues within the CBH-2 epitope would appear in escape variants from CBH-2 co-culture passaged virions. Any mutations that appeared in the R04 passaged virions would be considered as spontaneous mutations and would be ignored if they appeared in the variants from HC33.1 co-culture passages. At each passage of extracellular virus, infected cells were monitored for virus escape by screening with a two-color indirect immunofluorescence assay (IFA) that used both the test antibody, and a second antibody that recognized virus replication regardless of a change in envelope antigenicity (Fig. 2A) [28], [32]. In this case, cells infected with an escape variant were detected by a decrease or a loss of specific binding by the test antibody, HC33.1, but with retained binding by an anti-NS3 antibody. When escape was detected, RNA from escape variants was extracted from either cells or culture supernatants, reverse-transcribed, PCR amplified, and subcloned. Genomic residues 1491–2579 spanning the entire E2 coding region were sequenced from selected individual clones. The number of clones that were sequenced and analyzed ranged from 20 to 40 per sample. To ensure that newly released variants were transferred successfully to the next higher antibody concentration, repetitive passages of extracellular virions in the supernatant occurred at the lower antibody concentration until the percent of infected cells reached >80%. When passaged virions in supernatant resulted in >80% infected cells, the virus titers were usually >10^4^ FFU/ml (data not shown) [28], [32]. HCV genetic evolution under HC33.1 selection was analyzed and displayed after elimination of spontaneous mutations observed with R04 passaged virus (Fig. 2B and 2C). The passage numbers shown in Fig. 2 represent the evolving viral population and those passages having similar distribution of variants were not shown. New variants were observed and can be roughly separated into four phases under increasing concentrations of HC33.1. Because R04 has no effect on HCV, the concentration was raised rapidly and only 1–2 passages at each antibody concentration were needed to reach >80% infected cells. Spontaneous mutations located at V402A, N415D, N417S and F650Y were identified (Table S1), as previously reported, but with the addition of another mutation at 650 [28]. CBH-2 escape variants were isolated that contained the same mutations at two contact residues at 431 and 439, as previously reported (data not shown) [28], [32].

### Phase I (P0–P9)

To prepare sufficient virus stock, 2a JFH1 HCVcc was passaged multiple times in Huh7.5 cells. The final virus stock (P0) contained a mixture of wt (20%) and the glycan shifted N417S variant (80%). Nearly 100% of Huh7.5 cells infected with this stock was stained strong positive (+++) by both HC33.1 and anti-NS3 by IFA (upper panels in Fig. 2A and 2B, P0). Similar staining was observed with a control antigenic domain B antibody, CBH-5 [27], [47] (lower panels in Fig. 2A and 2B). Approximately 10^4^ FFU/ml of this stock was co-cultured initially with 0.25 µg/ml HC33.1 and designated as P1. After several passages of extracellular virions were collected from the supernatant, the percentage of infected cells was checked and found to be >80%. The antibody concentration was increased to 0.5 µg/ml and after three passages, at P6, the percent infected cell was again >80% and stained +++ with both HC33.1 and anti-NS3. Sequence analysis of extracellular virions revealed that the viral population shifted completely back to wt HCVcc with the glycan located at N417 (Fig. 2B). The viral population remained essentially wt for the next three passages, P6–9, as the antibody concentration increased to 1.0 µg/ml. The N-glycan shift from 415 back to 417 was a specific response to HC33.1 immune selection in that this change was not observed with the positive selection control, CBH-2, nor with the isotype-matched negative control, HMAb R04 (data not shown). Moreover, this is consistent with the greater sensitivity of the N417S variant to be neutralized by HC33.1 (Fig. 1B and 1C) that resulted in the re-emergence of wt HCVcc.

### Phase II (P10–P19)

When the antibody concentration increased from 1.5 to 4.5 µg/ml HC33.1 during P10–P19, a modest decrease in HC33.1 binding from +++ to ++ was observed by IFA (as shown for P18, Fig. 2A and 2B). CBH-5 staining by IFA remained unchanged at +++. Sequence analysis of extracellular virions at P10 (Fig. 2B) showed wt HCVcc decreasing to 30% and the appearance of four new variants, with each containing single or double mutations: N417T (40%), K610R (10%), N417S+K610R (10%) or N417T+N434D (10%). Although an N-glycan shift at N417S or N417T increased viral sensitivity to HC33.1 (Fig. 1B–D), the observed variants contained predominantly N417T-associated mutations as single, double or triple combinations with N434D and K610R. The following two passages, P12 and P13 (Fig. 2B), showed that wt HCVcc declined further to 10%, while the variant_N417T_ fluctuated between 20–40%. The most notable viral population change was the appearance of two new variants containing double or triple mutations, N417T+K610R or N417T+N434D+K610R, that replaced variant_N417S+K610R_ and variant_K610R_. The triple mutation containing variant_N417T+N434D+K610R_ emerged as the dominant strain from P14 to P19. At P18 and P19 (not shown), this variant accounted for 90% of the viral population (Fig. 2B). Taken together, it is possible that the N434D and K610R mutations provided some degree of survival benefit to the virus in combination with N417T and not with N417S. The N434D and K610R mutations are outside of the HC33.1 epitope that is located at aa 412–423 on E2. The increasing dominance of the triple mutation variant_N417T+N434D+K610R_ indicates that a variant having a N417T change requires additional mutations to provide some degree of protection from the neutralizing antibody, since N417T alone leads to a variant that is more sensitive to neutralization by HC33.1.

### Phase III (P20–P27)

During P20–P27 with concentrations of HC33.1 increasing from 5 to 6.0 µg/ml, the IFA intensity for HC33.1 binding decreased further from ++ to +, while CBH-5 staining remained +++ (as shown for P27, Fig. 2A and 2C). Sequence analysis showed two distinct changes. First, the triple mutation variant_N417T+N434D+K610R_ declined from 90% at P20 to 20% at P25 and eliminated at P27 (Fig. 2C). When HC33.1 increased from 5 µg/ml at P20 to 5.5 µg/ml at P21, the infected cell percentage rapidly decreased from 90% to 10%. Over the next four passages, this antibody concentration was maintained and the infected cell percentage gradually increased from 10 to 90% at P25. From P26 to P27, the infected cells remained high at 90%, even though HC33.1 was increased to 6.0 µg/ml. Second, a new mutation developed at S419N in mainly a quadruple mutation variant_N417T+S419N+N434D+K610R_. This variant increased from 10% at P20 to 70% at P27 (Fig. 2C). A second variant_N417T+S419N_ was also detected at a lower percentage of 10% at P27. The S419N mutation is within the HC33.1 epitope, although the residue at 419 is not a contact residue for the antibody (Fig. 1A). The findings suggest that the antibody concentration at P20 has risen to a level where the triple mutation variant_N417T+N434D+K610R_ can no longer survive. Increasing HC33.1 antibody concentrations led to greater selection pressure that resulted in the emergence of an S419N mutation. The location of the S419N mutation is between two contact residues for HC33.1 located at G418 and W420. Since W420 is also a contact residue for virus binding to CD81 [42], the S419N could lead to variants with diminished binding by HC33.1 and diminished binding by these variants to the HCV co-receptor.

### Phase IV (P28–P34)

From P27 to P29, an increase of HC33.1 from 6.0 to 6.5 µg/ml led to the appearance of two new variants with double, L413I+N417T (20%), and triple S395P+L413I+N417T (10%) mutations (Fig. 2C). For the first time, IFA analysis showed some infected cells staining positive by anti-NS3 but negative by HC33.1. Increasing HC33.1 concentrations more rapidly at 2.5 µg/ml increments from P29 to P30 (9.0 µg/ml) to P31 (11.5 µg/ml) led to rapid elimination of variants with the S419N mutation from 70% of combined variant_N417T+S419N+N434D+K610R_ and variant_N417T+S419N+N434D_ at P29 to 10% at P30, and their elimination at P31. During these passages of extracellular virions, infected cells remained at nearly 80% positive by anti-NS3 IFA staining but were completely negative by HC33.1 (as shown for P31, Fig. 2A). CBH-5 remained +++. Between P30 and P31, HC33.1 reached a critical concentration that eliminated S419N associated variants. It is probable that there are two contributing factors. First, repeated passages at concentrations ≥10 µg/ml reduced the proportion of S419N variants in the extracellular viral pool. Second, the L413I associated variants are able to enter Huh7.5 cells more efficiently leading to their rapid expansion. To confirm this possibility, extracellular virus from P31 was passaged once more at 11.5 µg/ml to increase virus stock and then placed in two high concentrations of HC33.1 at 20 (P33) and 50 µg/ml (P34) (Fig. 2C). As expected, virus infectivity remained high with nearly 90% of cells stained positive by anti-NS3 and negative by HC33.1. Sequence analyses of P33 and P34 were identical with 90% variant_S395P+L413I+N417T_ and 10% variant_L413I+N417T_.

### Mutations that are associated with progressive reduction in HC33.1 binding and neutralization

**Figure 3:**
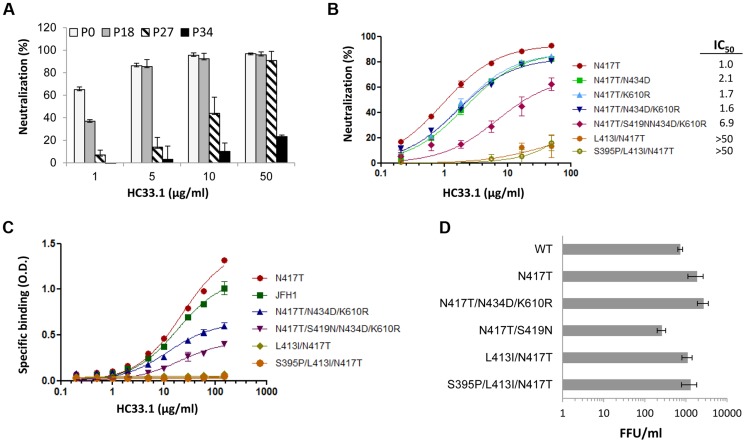
Analysis of escape variants on their sensitivity to HC33.1-mediated neutralization and binding, and their effect on in vitro viral fitness. (**A**) HC33.1 dose-dependent neutralization against viral pool in culture supernatants collected from P0, P18, P27 and P34 with their respective dominant variants bearing the following mutations: N417S, N417T/N434D/K610R, N417T/S419N/N434D/K610R and S395/L413I/N417T was performed by FFU-reduction assay. (**B**) Dose-dependent neutralization against recombinant HCVcc variants bearing specific mutations, as identified in each phase of viral escape selection was performed by SEAP reporter assay. The IC_50_ value against each variant is tabulated in the legend. (**A, B**) Each assay was performed in triplicates and data are shown as percent neutralization, the mean of two experiments ±SD. (**C**) HC33.1 binding to specific variants, as identified in the legend, by ELISA. Recombinant JFH1 E1E2 wt or the indicated variant E1E2 lysate was captured by GNA in microtiter wells. The wells were then incubated with HC33.1 at the indicated concentrations (0–150 µg/ml). Binding was detected after anti-human IgG-labeled horseradish peroxidase. The *y*-axis shows the mean optical density values for triplicate wells, the mean of two experiments ±SD. (**D**). Effect of specific mutations on in vitro viral fitness was determined by measuring virus yield of wt or variant HCVcc bearing the indicated mutations in the focus forming unit assay at an MOI of 0.1. Each assay was performed in triplicates and data are shown as FFU/ml, the mean of two experiments ±SD.

The sensitivity of escape variants isolated at different passages compared to the initial virus stock (consisting mainly of variant_N417S_ at P0) was tested in dose-dependent studies with HC33.1 (Fig. 3A). At P18 (phase II), when the dominant variant contained triple mutations, N417T/N434D/K610R, a modest reduction of 40% in neutralization sensitivity was apparent only at 1 µg/ml and not at the higher antibody concentrations. At P27 (phase III), when the dominant variant contained quadruple mutations, N417T/S419N/N434D/K610R, a more significant reduction of 50–75% in sensitivity to HC33.1 was observed at concentrations between 1–10 µg/ml. From P31–P34 (phase IV), when cells infected with passaged virions no longer showed HC33.1 binding by IFA and the dominant variant contained S395P/L413I/N417T mutations, essentially no neutralization was observed against passaged virus (P34) at all antibody concentrations. To verify these findings, and because wt HCVcc declined rapidly in the beginning of phase II and new variants appeared in combination with N417T, the variant_N417T_ was employed as the reference virus to determine the role of mutations observed in phase II (N434D and K610R), in phase III (S419N), and in phase IV (L413I). The N434D and K610R mutations were engineered with N417T in double and triple mutation bearing variants. The S419N mutation was engineered in single and quadruple mutation variants. For the two variants observed at P31–P34 having mutations at L413I, with or without S395P, both were constructed in the context of N417T. HCVcc plasmid DNA constructs were made and their corresponding viruses were harvested following electroporation of the viral RNA into Huh7 cells. The neutralization sensitivities of these recombinant HCVcc variants were then measured (Fig. 3B). Recombinant variant_N417T/N434D_ (IC_50_ 2.1 µg/ml), variant_N417T/K610R_ (IC_50_ 1.7 µg/ml) and variant_N417T/N434D/K610R_ (IC_50_ 1.6 µg/ml) had nearly two times the IC_50_ values as variant_N417T_ (IC_50_ 1.0 µg/ml). This indicated that N434D and K610R mutations contributed to modest decrease in neutralization sensitivity to HC33.1, although combining both mutations had no additive effect. For the S419N mutation observed in phase III, the variant_N417T/S419N/N434D/K610R_ showed a more substantial decrease in neutralization sensitivity with IC_50_ values of 6.9 µg/ml. The variants bearing mutation at L413I with or without the S395P mutation, observed in phase IV, were completely resistant to HC33.1 neutralization (>50 µg/ml). The findings with constructed HCVcc variants collectively confirmed the results observed with passaged virions obtained in phase II, III and IV. To assess that the drop in HC33.1 neutralization potency is due to decrease in antibody binding to these escape variants, binding studies against recombinant variant E1E2 cell lysates were performed (Fig. 3C). Binding by HC33.1 to the N417T variant was greater than to wt JFH1, which is consistent with greater neutralization potency against the N417T HCVcc variant (Fig. 1B and 1D). As expected, progressive decrease in binding was observed in the following order of variants: N417T>N417T/N434D/K610R>N417T/S419N/N434D/K610R. No binding was observed with either L413I/N417T or S395P/L413I/N417T associated variants, which is consistent with complete viral escape associated with the L413I mutation. To confirm that the S395P mutation had no effect on HC33.1, a variant having just S395P was tested and no reduction in HC33.1 or AP33 binding was observed (Fig. S1).

### Effects of escape mutations on in vitro viral fitness

The aa 412–423 region is known to be highly conserved and involved in virus binding to CD81 [42]. This implies that the region is under functional constraints and that viral escape from neutralizing antibodies to this region will be at least associated with compromised in vitro viral fitness. However, escape from AP33 occurs when there is an N-glycan shift from 417 to 415 [45] (Fig. 1B–ID). More importantly, the escape variants bearing either N417S or N417T mutation exhibited improved viral fitness (Fig. 1E). A similar viral escape pattern from HCV1 has been also documented in experimental animals and HCV infected patients [44], [46]. Since the viral escape pattern from HC33.1 is different from AP33, viral fitness of the dominant variants identified in phase II, III and IV were measured. To avoid possible contribution of non-E1E2 mutations contributing to fitness in passaged virus, the constructed recombinant HCVcc variants were employed for this study. Huh7 cells were infected with each variant HCVcc at a 0.1 MOI and the infectious virus yield at 96 hours was determined (Fig. 3D). From phase II, the two mutations, N434D and K610R in the context of N417T, variant_N434D+N417T+K610R_ had a higher virus yield compared to wt HCVcc (*P* = <0.003). The S419N mutation observed in phase III in the context of N417T, variant_N417T+S419N_, had nearly three-fold decrease in virus yield compared to wt HCVcc (*P* = <0.017). The L413I mutation in variant_L413I+N417T_ and variant_S395P+413I+N417T_ had modest reductions in virus yield compared to N417T, but they were not significantly higher than wt HCVcc (respective *P* values = <0.177 and  = <0.106). All escape variants essentially had higher or normal in vitro viral fitness except for the variants with the S419N mutation having a compromised viral fitness.

### Stable and unstable mutations associated with progressive escape

**Figure 4:**
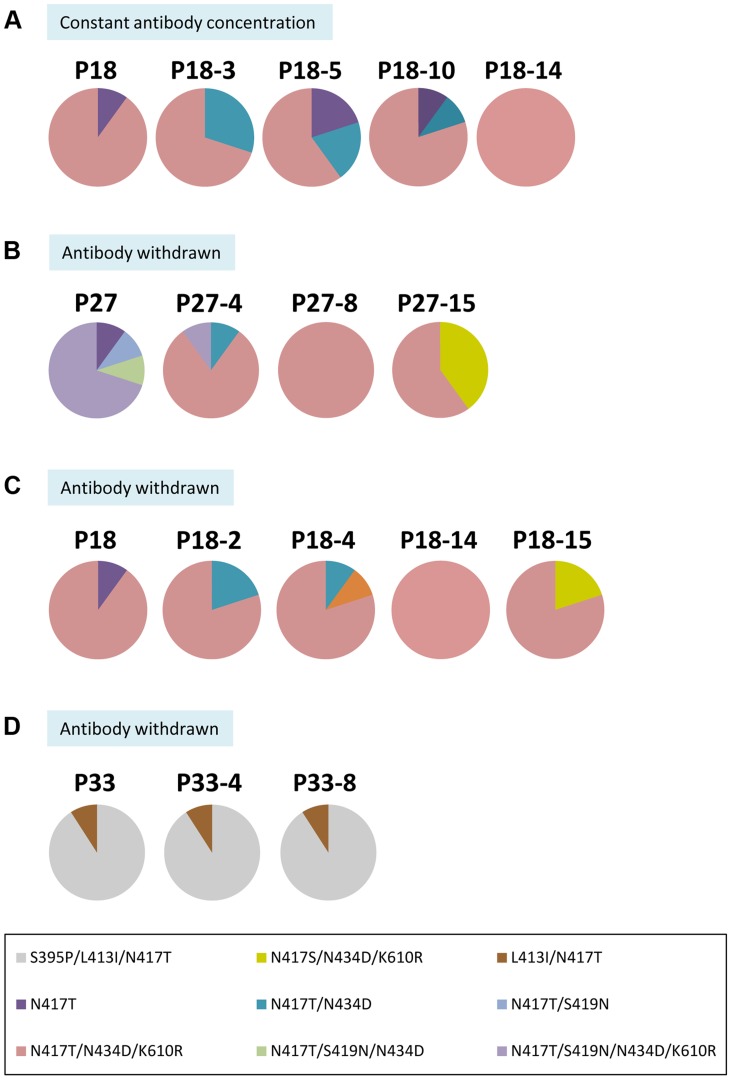
Dose-dependent escape variants and their stability with and without continuing immune pressure. (**A**) The composition of the viral pool at P18 containing the dominant variant_N417T+N434D+K610R_ during five additional passages at a constant concentration of 4.5 µg/ml HC33.1. (**B**) The composition of the viral pool at P27 containing the dominant variant_N417T+S419N+N434D+K610R_ during 15 additional passages after HC33.1 was removed. (**C**) The composition of the viral pool at P18 containing the dominant variant_N417T+N434D+K610R_ during 15 additional passages after HC33.1 was removed. (**D**) The composition of the viral pool at P34 containing the dominant variant_S395P+L413I+N417T_ during 8 additional passages after HC33.1 was removed. (**A–D**) The identified specific variants are color coded as indicated in the legend.

The S419N mutation emerged at a critical inflection point of 5 µg/ml HC33.1 (P20) and not at 4.5 µg/ml (P18) (Fig. 2C). This raised a question whether the S419N substitution is a truly concentration-dependent mutation. To address this possibility, the extracellular virus pool at P18, which did not contain S419N associated variants, was passaged 14 more times with the HC33.1 concentration remaining constant at 4.5 µg/ml (Fig. 4A). Sequence analysis showed that the triple mutation variant_N417T/N434D/K610R_ persisted as the dominate isolate with other N417T variants, variant_N417T/N434D_ and variant_N417T_, at lower percentages in the viral pools and eventually not detected (as shown for P18-3 to P18-14, Fig. 4A). The fact that the S419N mutation did not emerge indicated that the variant containing triple mutations, N417T+N434D+K610R, sufficiently altered HC33.1 binding and neutralization such that the virus can persist under continuous immune selection at this antibody concentration. When the virus was first exposed to 5 µg/ml HC33.1 at P20, the first indication of an S419N mutation was observed and the variant_N417T/S419N/N434D/K610R_ rapidly expanded over subsequent passages (Fig. 2C). To further prove that the S419N mutation is concentration dependent, HC33.1 was withdrawn from and repeatedly passaged to determine whether the S419N mutation reverted back to the wt residue at this position. The P27 viral pool comprising 70% of variant_N417T/S419N/N434D/K610R_ was passaged 15 more times in the absence of HC33.1 (Fig. 4B). Sequence analysis showed that the variant_N417T/S419N/N434D/K610R_ reduced from 70% to 10% in the first 4 rounds and completely disappeared in the following rounds. The dominant isolates at P27-8 to P27-15 were triple mutation variant_N417T/N434D/K610R_. At P27-15, an N417S associated variant_N417S/N434D/K610R_ appeared. The pattern shows that the induction of the S419N mutation is HC33.1 antibody concentration dependent and occurred in the context of the triple mutation variant_N417T/N434D/K610R_. It is possible that these mutations influenced the emergence of the S419N mutation.

To determine whether the triple mutation variant_N417T/N434D/K610R_ is stable, the viral pool at P18, containing 70% variant_N417T/N434D/K610R_, was passaged repeatedly without HC33.1 (Fig. 4C). Sequence analysis showed that this variant persisted. It should be noted that when HC33.1 was withdrawn from P18 and P27 (Fig. 4B and 4C), the adaptive mutation at N417S eventually returned and co-existed with N417T. This provides additional proof that the N417T mutation is a specific response to HC33.1 immune selection. The L413I mutation is associated with stable variants (Fig. 4D). When the P34 viral pool was passaged eight times without HC33.1, sequence analyses remained the same throughout these passages, consisting of 90% variant_S395P/L413I/N417T_ and 10% variant_L413I/N417T_. Overall, stable and unstable mutations were induced under antibody pressure. The mutations at 417 and 434 are a direct response to low antibody pressure without a cost in viral fitness. Consequently, when antibody pressure is withdrawn, variants containing these mutations will persist. The mutation at 419 leads to more resistant variants having compromised fitness. When antibody pressure is withdrawn, the virus reverts back to a wt residue at this position that restores an improved fitness.

## Discussion

These studies defined the pathways of viral escape and the formation of quasispecies from a single neutralizing antibody directed against a conserved region encompassing aa 412–423 on the E2 glycoprotein that underscore the difficulty in vaccine design for this highly variable virus. Neutralization escape occurred in a nonrandom stepwise progression in response to the antibody concentration and was mediated by multiple mechanisms with relatively few amino acid changes. The mutations were at residues within and outside of the region encompassing the epitope, and some were associated with a N-glycan shift. Multiple variants appeared when infectious virions were co-cultured with low antibody concentrations that have two mutations, N434D and/or K610R, located outside of the epitope. These variants were stable and not associated with reduced in vitro viral fitness. Their development exemplifies the formation of variants or quasispecies from one antibody that contributes to viral persistence in the presence of neutralizing antibodies. In contrast, mutations within the region encompassing the epitope, aa 412–423, had different effects. The N-glycan shift associated mutation at S419N reverted to wt residue at this position when antibody selection pressure was lowered or withdrawn. The S419N associated variants, having compromised in vitro viral fitness, highlight the constraints on molecular evolution within the epitope because of the essential role of this region in HCV entry. A third mutation at a contact residue, L413I, occurred at higher HC33.1 selection pressure that resulted in variants completely escaping virus neutralization by this antibody. These variants were stable and had in vitro viral fitness similar to wt HCVcc. Although the L413I mutation was elicited in these studies, the Leu in this position is highly conserved in patient sequences [45]. Only 11 of 2108 curated E2 sequences of >100 bases length in the LANL Hepatitis C Virus Database, varied from Leu at this position [48], [49]. All of these variants are L413P, except for a genotype 5a sequence from South Africa which is L413F. Additionally, only 18 of 25629 uncurated E2 sequences deposited in GenBank since 2009 varied from Leu at 413. Again all of these variants were L413P, except for one each of L413Q, L413F, L413H and L413V. The elicited L413I mutation is therefore considered not to be found thus far in nature. Similarly, in a subset of the 2108 LANL HCV Database sequences consisting of 1311 longer high-quality E2 sequences, Gly at 418 is highly conserved and only two out of these 1311 sequences varied from Gly. The Trp at 420 is absolutely conserved. Only one of the 1311 sequences contained Trp to Arg change at 420 and this is more likely due to PCR error since the W420R mutation is not tolerated in JFH1 HCVcc (unpublished data). The observed conservation of Leu at 413 raises a question why the L413I mutation has not been documented more frequently in light of our studies and because the L413I associated variants are stable, and without compromised fitness. One possible explanation is that HC33-like antibodies are of low frequency [33], [34] and when present are of low titers. The low immunogenicity of aa 413–423 is possibly due to the masking effect of HVR1 [50]. Consequently, the L413I mutation is not necessary for HCV infection to persist in the majority of HCV infected individuals. Another possibility is that this mutation is strictly an in vitro virus-antibody co-culture phenomenon. Overall, the combination of multiple escape pathways enables the virus to persist under a wide range of antibody concentrations.

In previous studies with antigenic domain B antibodies, epitope mapping by alanine scanning revealed that their cognate epitopes were located in two discontinuous segments on E2, encompassing aa 425–443 and aa 529–535 [32], [51]. The region at 529–535 is highly conserved and under functional constraints, because these residues participate in E2 interaction with CD81 [42]. The 425–443 region located immediately downstream of the 412–423 region is a more variable region and five of these residues at positions 431, 434, 435, 438 and 439 are sites of escape mutations from domain B antibody-mediated neutralization [28], [31], [32]. Among the five residues, substitutions at 431 and 439 have no negative impact on in vitro viral fitness [28], [32]. But the other three at 434, 435 and 438 adversely affect fitness by reducing virus binding to CD81 [32]. In the current studies, partial escape from HC33.1 at low antibody concentrations also involved the 434 residue, although this antibody is against a linear epitope upstream of this location. A mutation at 434 reduced antibody binding and neutralization. These observations taken together outline a functional sequence organization at the N-terminal end of E2. It consists of a conserved region at aa 412–423 that is flanked by two variable regions, the HVR1 located at aa 383–411 and a second variable region located at 425–443. While mutations within HVR1 are mostly in response to antibodies directed at linear epitopes within HVR1, the 425–443 variable region is responsible for escape from antigenic domain B antibodies and now, antigenic domain E antibodies (as represented by HC33.1). It is possible that the K610R mutation is indicative of a different variable region on E2. In other studies, we found that the C-terminal end of this 425–443 variable region, encompassing 441–443, is actually quite conserved. These three residues form a critical binding pocket of a cluster of overlapping epitopes, designated as antigenic domain D [28], [52]. HMAbs directed at domain D are not likely to be associated with viral escape. Taking this into consideration, the variable region is more restricted to the region encompassing aa 425–440.

The second pathway of escape involves mutations associated with a glycan shift. Asparagine (N-) linked protein glycosylation plays crucial roles in viral protein folding and in regulation of protein functions that include epitope accessibility. The most commonly used glycosylation sequon was first defined as Asn-X aa-Ser/Thr (Xaa Pro) [53], and since this first observation, more variable sequons have been reported (review in [54]). In the aa 412–423 sequence on E2, there are three residues, N415, N417 and S419, perfectly placed for a “glycosylation sequon” that allows a glycan shift either forward (+2) or backward (−2) [55]–[57] with the N417 as the key residue. Through the studies of HCV evolution in the presence of HC33.1, at least four escape strategies are linked to these three residues that impact antibody access to its epitope and affecting the neutralization potency of the antibody. However, some of these escape mechanisms are associated with a cost in fitness. First, Asn at the 417 position, designated as the first N-glycan among twelve in the highly glycosylated HCV E2 glycoprotein, is highly conserved among HCV genotype and subtype isolates [18]–[21]. The position of this N1 glycan appears to have a greater negative modulating effect on HC33.1 than AP33, with both directed at aa 412–423 (Fig. 1B–1D). This is supported by the observed shift in viral population from predominantly N417S in the virus stock (P0) back to a more uniform wt population, as soon as the virus was exposed to HC33.1, albeit at a low concentration. The reappearance of wt JFH1 HCVcc was the first step in viral escape because wt virus is more resistant to HC33.1 neutralization than the N417S variant (Fig. 1B and 1C).

Second, the glycan shift associated with the N417S mutation occurs spontaneously, as observed in natural infection [44], [46] and in passaged cell culture HCVcc [45]. The N417S change is an adaptive mutation that leads to a variant with greater in vitro viral fitness (Fig. 2E) and is able to completely escape from AP33, but not the HC33 antibodies to aa 412–423 (Fig. 1C). The implication is that AP33 and the HMAb HCV1 [44]–[46] targeting the same region as HC33.1 are more glycan-dependent.

Third, both Ser and Thr are believed to be equal alternative amino acids in glycosylation sequon (Asn-Xaa-Ser/Thr). However, the selection of Thr substitution (Asn-Xaa-Thr) is associated with HC33.1 immune pressure and not Ser substitution (Asn-Xaa-Ser) (Fig. 2B and 2C). The finding that N417S associated variants began to be detected after withdrawal of HC33.1 from passaged virus supports our analysis of a Thr substitution specifically induced by this antibody (Fig. 4B and 4C). The reason why Asn-Xaa-Thr was preferentially selected in the presence of antibody is not entirely clear because N417T HCVcc and N417S HCVcc have similar fitness and sensitivity to HC33.1 (Fig. 1). Some studies noted that glycosylation of Asn-Xaa-Thr sequons is approximately 40 times more efficient than that of Asn-Xaa-Ser sequons [58], [59]. In addition, the selection of Asn-Xaa-Thr over Asn-Xaa-Ser occurred in response to an antibody directed at the aa 412–423 region but not by neutralizing antibodies directed at other antigenic regions. When escape studies were performed with antigenic domain B antibodies, the N417T mutation has not been observed [28], [32]. Importantly, during viral evolution in the presence of HC33.1, the N434D, K610R and S419N escape mutations occurred in the context of N417T and not N417S. Taken together, the N417T change is a specific response to HC33.1 and not an adaptive mutation. The identification of the N417T mutation provides additional support that the virus-antibody co-culture studies with 2a HCVcc is applicable to escape studies with other HCV genotypes/isolates. Clinical studies with HMAb HCV1 in liver transplant recipients infected with genotype 1a led to escape variants having the N417T or N417S mutations [44].

Fourth, it is possible that the S419N mutation generated a new glycan at 419 using −2 glycosylation sequon (Thr**-**Xaa-Asn) [55]–[57]. A shared element between N1 and S419N-associated glycans is that the middle residue in both sequons is G418, a contact residue for HC33.1. Antibody access to this residue will be blocked by either glycans. The S419N glycan is more efficient than the N1 glycan in shielding the HC33.1 and resulting in a significant drop in antibody binding and neutralization (Fig. 3A–3C). However, the formation of the S419N glycan is associated with compromised in vitro viral fitness, which can be attributed to this glycan being in closer proximity to W420, a contact residue for virus binding to CD81 [42]. The effect of S419N glycan shift in reducing HC33.1 binding explains the persistence of variants with compromised fitness over the course of multiple passages (P20–P29) under continuous immune selection. Furthermore, the cost in viral fitness associated with S419N explains why the N434D mutation occurred first in the context of N417T because the fitness of these variants was not significantly compromised. But the ability of this mutation to reduce HC33.1 binding was not as significant as S419N.

The transition from nearly uniform wt virions (P6–P9) to variants that were more able to co-exist with low levels of HC33.1 in phase II (P18) (Fig. 2B) appears to be a non-random process during viral evolution. There were a restricted number of mutations at a limited number of sites during sequence space expansion (P10) and contraction (P13–P14). All four variants had a combination of N417T, N434D and K610R mutations at P13–14 that were the same mutations in the dominant variant_N417T/N434D/K610R_ at P18. In this phase of viral escape, a mutation occurred in a region that is more variable, e.g., 425–440, and not under functional constraints. Mutations in this variable region are more likely to be in stable variants that persist regardless of continuous presence of neutralizing antibody (Fig. 4A) or absence of antibody (Fig. 4C). The development of stable variants or quasispecies that are more resistant to virus neutralization partly explains why HCV co-exists with neutralizing antibodies during chronic infection. While this balance between variants having robust fitness and antibody can be maintained in repeated passages (Fig. 4A), it can be disrupted by a slight increase in antibody concentration. When HC33.1 reached 5 µg/ml at P20 (Fig. 2C), a quadruple mutation emerged precisely by the addition of the S419N mutation onto the existed triple mutation variant, without undergoing a sequence space expansion. The quadruple mutation variant preferentially replicated over the triple mutation variant (from P20 to P27). Although the quadruple mutation variant_N417T/S419N/N434D/K610R_ can co-exist at higher levels of HC33.1 (5.0–6.0 µg/ml), viral escape is still incomplete since the virus can be neutralized at a higher percentage at higher antibody concentrations (Fig. 3A and 3B). This suggests that incomplete escape is sufficient for the virus to co-exist with HC33.1.

The antibody concentration-dependent stepwise escape pattern is suggestive of low affinity or low antibody concentration facilitating the formation of escape variants or quasispecies. This could be the scenario during acute HCV infection when a wide range of lower affinity neutralizing antibodies is more likely to be elicited. As acute infection progresses to persistent infection, a more robust neutralizing antibody response provides immune pressure that leads to the selection of escape variants with compromised fitness. This in turn will contribute to a transient reduction in viral load resulting in a reduced B cell response. The lowering of a neutralizing antibody response leads to the release of virions with greater fitness but potentially greater sensitivity to virus neutralization, as observed when the S419N mutation changed back to wt when the neutralizing antibody was removed. This cycle may explain in part persistent viremia during chronic HCV infection in the presence of serum neutralizing antibodies. At the same time, our findings pose a different challenge to vaccine development beyond the identification of highly conserved epitopes mediating virus neutralization. It will be necessary to induce high potency neutralizing antibodies to multiple epitopes within 412–423 that prevent the formation of escape variants, which can co-exist with lower potency or levels of neutralizing activities. The fact that the aa 412–423 segment on E2 is the target of both AP33-like and HC33.1-like antibodies increases the importance of this region in an effective HCV vaccine. It will be more difficult for the virus to escape simultaneously from both sets of these antibodies. The isolation and characterization of neutralizing human monoclonal antibodies to HCV will further our understanding of viral neutralization escape mechanisms that will be necessary for vaccine design.

## Materials and Methods

### Ethics statement

Ethical approval was obtained from the Administrative Panel on Human Subjects in Medical Research (protocol number 13860), Stanford University, Stanford, California, USA. Written informed consent was obtained from the participant.

### Cell culture, antibodies, virus, and reagents

HEK-293T cells were obtained from the ATCC. Huh7 [60] and Huh7.5 cells, generously provided by Dr. Charles Rice (Rockefeller University), were cultured at 37°C, 5% CO_2_ in DMEM (Invitrogen, Carlsbad, CA) supplemented with 10% fetal calf serum (FCS) and 2 mM glutamine (Sigma-Aldrich Co., St. Louis, MO). The secreted alkaline phosphatase (SEAP) reporter cell line Huh7J-20 was described previously [61]. HMAbs HC33.1, CBH-5, CBH-17 and HC-11 against HCV E2 have been described previously [25], [47], [51]. A MAb against HCV NS3 protein was generously provided by Dr. George Luo (University of Kentucky). JFH-1 2a virus was generously provided by Dr. Takaji Wakita (National Institute of Infectious Diseases, Japan). Virus stocks were produced as described in [51], [62] and virus titers were determined by a focus-forming unit assay, FFU, as described [32].

### Isolation of variants escaping antibody-mediated neutralization

JFH-1 2a HCVcc was employed in this study to determine viral evolution under HMAb HC33.1 and performed essentially as described [28], [32]. Briefly, Huh7.5 cells (3.2×10^4^/ml) seeded 24 hrs previously in a 24-well plate were inoculated with a mixture of HCVcc (1×10^4^ FFU) and HMAb HC33.1. The initial concentration of the neutralizing antibody was adjusted to the 25% inhibitory concentration (0.25 µg/ml) of the antibody against the 2a HCVcc. HMAb anti-CMV R04 was used as mock human IgG selection. The cells were collected for analysis by indirect immunofluorescent assay (IFA) and the extracellular virus was harvested for virus titration, the next passage of selection, and for viral sequence analysis. The entire process constituted one passage of infectious virus. To ensure that minority variants have a high probability to be passed to the next round selection, extracellular virions were repeatedly passaged until the virus titer reached 1×10^4^ FFU/ml, which correlated to ≥80% infected cells. Growth of extracellular virus was measured by FFU assay and the emergence of escape variants was monitored weekly by two-color confocal immunofluorescence microscopy and by staining with HC33.1 and an anti-NS3 antibody. To assess the relationship between emerging specific mutations and the antibody concentration, selected viral supernatants were passaged in the growth medium containing antibody at a fixed concentration or no antibody (antibody was withdrawn from the medium) for a number of rounds as indicated.

### Viral sequencing analysis

Total RNA or viral RNA from virus-containing culture supernatant was extracted using commercial kits (Qiagen, Valencia, CA) and reverse transcribed to cDNA SuperScript III reverse transcriptase (Invitrogen, Carlsbad, CA) using primer p7rev (CCCGACCCCTGATGTGCCAAGC). The envelope genes (E1E2) were amplified using the Expend High Fidelity PCR system (Roche Applied Sciences, Indianapolis, IN) and primers E1F (GGAACCTTCCTGGTTGCTCTTTCTCTATCTTCC) and E2R (TGCTTCGGCCTGGCCCAACAAGAT). The PCR products were ligated into the Topo cloning vector (Invitrogen, Carlsbad, CA), and individual clones containing an insert of the expected size were sequenced in both sense and antisense strands (Elim Biopharm, Hayward, CA). Selected PCR products were cloned into pCDNA 3.1 expression vector for protein production in binding assay.

### Virus neutralization

Neutralization against extracellular virus in cultured supernatant from different passages was measured by FFU-reduction neutralization assay as previously described [28]. The antibody concentration causing 50% reductions in FFU was determined by linear regression analysis. The percent neutralization was calculated as the percent reduction in FFU compared with virus incubated with an irrelevant control antibody. Neutralization against recombinant virus variants were performed using Huh7J-20 cells, and virus infectivity levels were determined by SEAP reporter assay, as described previously [61]. Briefly, Huh7J-20 cells were plated out 24 hrs prior to infection at a density of 3×10^3^ per well in a 96-well plate. Virus was pre-incubated at 37 C for 1 h with the appropriate antibody prior to infecting the cells at an MOI of 0.1. At 3 h post-infection, the inoculum was replaced with fresh medium and incubated for 72 hrs. The virus infectivity levels were determined by measurement of the SEAP activity released into the medium.

### Quantitative enzyme-linked immunoassay

ELISA was performed to measure antibody binding to the wt or mutant E2 glycoproteins, as described [28]. Briefly, microtiter plates were prepared by coating each well with 500 ng of Galanthus nivalis agglutinin (GNA) and blocking with 2.5% nonfat dry milk and 2.5% normal goat serum. Lysates of cells expressing wt HCV, mutant E1E2, or pelleted virus were captured by GNA on the plate and later bound by a range of 0 to 150 µg/ml of HMAb. The bound HMAb was detected by incubation with alkaline phosphatase-conjugated goat anti-human IgG (Promega; Madison, WI), followed by incubation with *p*-nitrophenyl phosphate for color development. Absorbance was measured at 405 nm and 570 nm.

### Epitope mapping

Epitope mapping was performed using alanine substitution mutants of a defined E2 region: aa 411–424. Alanine substitution mutants were constructed in plasmids carrying the 1a H77C E1E2 coding sequence (GenBank accession number AF009606) as previously described [28]. All the mutations were confirmed by DNA sequence analysis (Sequetech, Mountain View, CA) for the desired mutation and for exclusion of unexpected residue changes in the full-length E1E2 encoding sequence. The resulting plasmids were transfected into HEK293T cells for transient protein expression using the calcium-phosphate method. The mutated constructs were designated X#Y, where # is the residue location in H77C, X denotes the single-letter code for the H77C amino acid, and Y denotes the altered amino acid.

### Virus yield assay as a measure of in vitro viral fitness

Virus-containing supernatants were inoculated onto Huh7 cells at a multiplicity of infection (MOI) of 0.1. The cells were seeded 24 h previously in a 24-well plate. After 3 h of incubation at 37°C and 5% CO_2_, the inoculum was replaced with fresh complete medium and incubated for an additional 96 hrs. The supernatant fluids were then collected, and the titer of infectious virus was assessed by the SEAP reporter assay, as described previously [61].

### Site-directed mutagenesis

To evaluate contribution of mutated individual amino acid observed during viral evolution, introduction of amino acid change was conducted using a QuikChange II site-directed mutagenesis kit as described previously [28], [32]. All the mutations were confirmed by DNA sequence analysis (Sequetech, Mountain View, CA) for the desired mutation and for exclusion of unexpected residue changes in the full-length E1E2-encoding sequence. The mutated constructs were designated X#Y, where # is the residue location in H77C, X denotes the single-letter code for the H77c amino acid, and Y denotes the altered amino acid. The exception is for the K610, where # (610) is the residue location in JFH1 that corresponds to # (606) residue location in H77C.

### Statistical analysis

Statistical analyses were performed using unpaired Student *t* test (GraphPad software), with p values<0.05 considered statistically significant.

### Bioinformatics analysis

Sequences were downloaded from the Los Alamos National Laboratory Hepatitis C Database (http://hcv.lanl.gov/content/index, [48], [49]), which contains a set of curated sequences deposited prior to 2009. E2 sequences deposited in GenBank (http://www.ncbi.nlm.nih.gov) from 2009 onwards were also retrieved. Alignments were made and viewed in MEGA6 (www.megasoftware.net [48], [49].

## Supporting Information

Figure S1 **Dose-dependent binding of HC33.1 to a variant bearing S395P mutation.** HC33.1 and AP33 binding to variant_S395P_, by ELISA. Recombinant variant E1E2 lysate was captured by GNA in microtiter wells. The wells were then incubated with HC33.1 or AP33 at the indicated concentrations (0–150 µg/ml). Binding was detected after anti-human or anti-mouse IgG-labeled horseradish peroxidase. The *y*-axis shows the mean optical density values for triplicate wells, the mean of two experiments ±SD.
(TIF)

Table S1 **Percent spontaneous mutations in the presence of R04, an isotype-matched control.** R04 is an IgG_1_ HMAb to HCMV, employed as an isotype-matched control. Table outlines mutations with their corresponding frequency that were identified during different passages as the antibody-concentration was increased to 50 µg/ml.
(TIF)
