# Supplementary figures and images for: Non-random Escape Pathways from a Broadly Neutralizing Human Monoclonal Antibody Map to a Highly Conserved Region on the Hepatitis C Virus E2 Glycoprotein Encompassing Amino Acids 412–423
Source: PLoS Pathog. 2014 Aug 14;10(8):e1004297. doi: 10.1371/journal.ppat.1004297 (PMC4133389; doi:10.1371/journal.ppat.1004297)

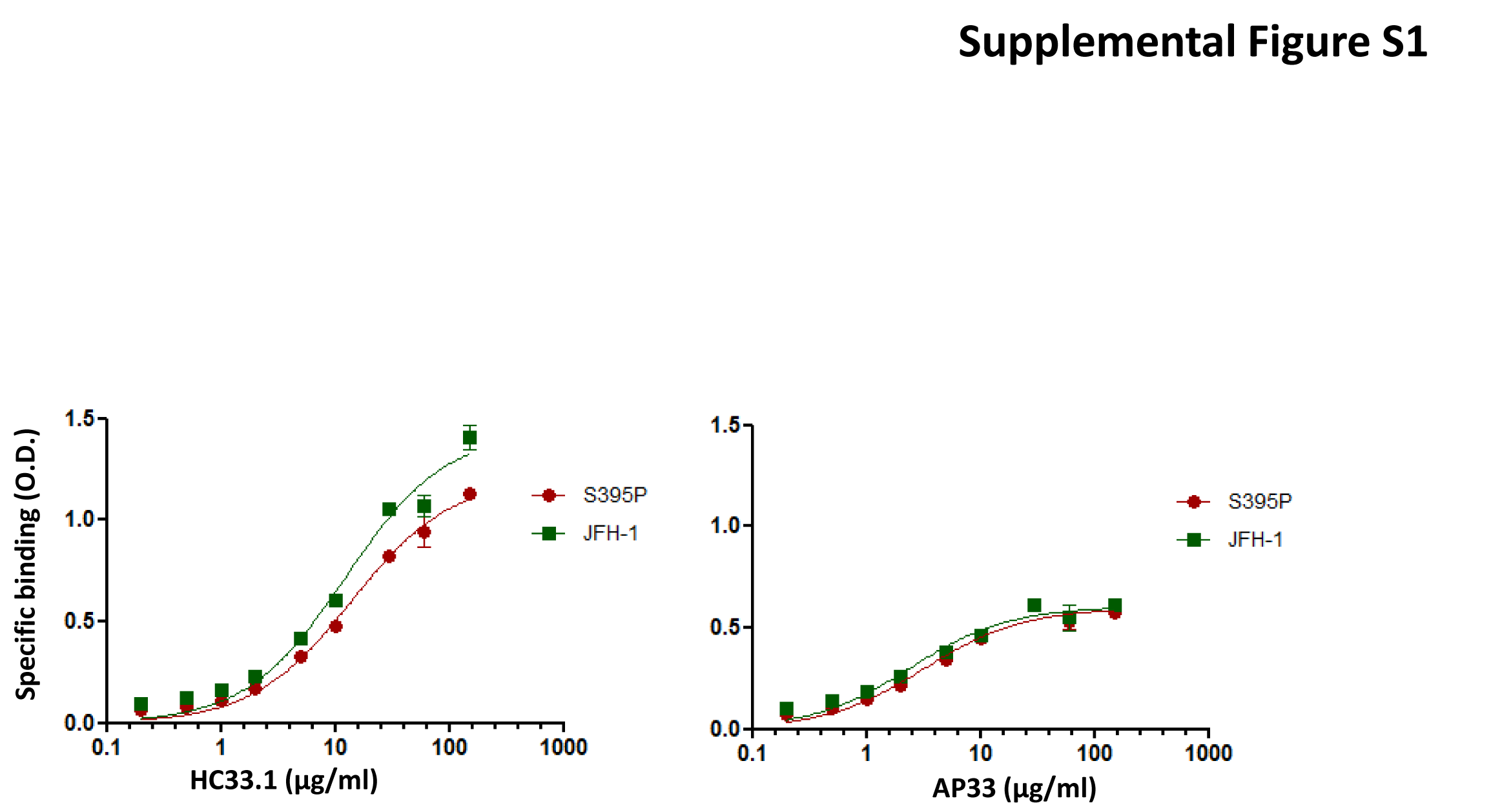

Supplement: Figure S1 — Dose-dependent binding of HC33.1 to a variant bearing S395P mutation. HC33.1 and AP33 binding to variantS395P, by ELISA. Recombinant variant E1E2 lysate was captured by GNA in microtiter wells. The wells were then incubated with HC33.1 or AP33 at the indicated concentrations (0–150 µg/ml). Binding was detected after anti-human or anti-mouse IgG-labeled horseradish peroxidase. The y-axis shows the mean optical density values for triplicate wells, the mean of two experiments ±SD. (TIF) [file ppat.1004297.s001.tif]

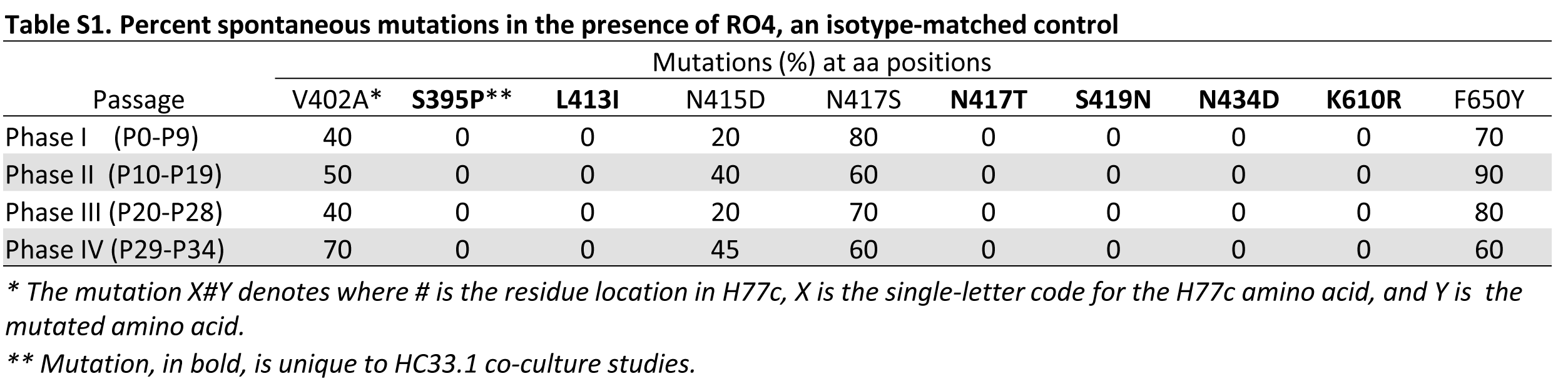

Supplement: Table S1 — Percent spontaneous mutations in the presence of R04, an isotype-matched control. R04 is an IgG1 HMAb to HCMV, employed as an isotype-matched control. Table outlines mutations with their corresponding frequency that were identified during different passages as the antibody-concentration was increased to 50 µg/ml. (TIF) [file ppat.1004297.s002.tif]
